# Reviewed article: Nascimento, ADF; Souza, MCL; Rossoni, SL; Santos, JH; Lopes, WA; Pujals, C; Pedroso, RB. Integrative and complementary practices, hypertension and social indicators: an ecological study, Brazil, 2023. Epidemol Serv Saude. 2026:35;e20250570

**DOI:** 10.1590/S2237-96222026v35e20250570.b

**Published:** 2026-10-05

**Authors:** Ana Elisa Madalena Rinaldi

**Affiliations:** 1 Universidade Federal de Uberlândia, Uberlândia, MG, Brazil

## First round comments

O presente manuscrito recebeu as avaliações dos pareceristas, as quais estão descritas no final deste e-mail. É importante destacar que além dos pareceres técnicos - relacionados ao conteúdo metodológico - também foi emitido parecer referente à padronização do manuscrito segundo as normas da RESS e Similaridade do texto.

Os pareceres destacaram principalmente os seguintes aspectos:

- Revisão do resumo, especialmente quanto aos métodos (necessidade de citar quais as variáveis foram selecionadas no SISAB e a análise estatística) e às conclusões (ajuste para conclusões que podem ser sustentadas pelos resultados encontrados).
- Ajuste do objetivo do estudo - fazer alinhamento entre o objetivo descrito no resumo, na introdução e especialmente ao longo do manuscrito a fim de ficar claro sua finalidade principal
- Métodos - é necessário especificar e detalhar quais os indicadores referentes à hipertensão arterial e aos dados sociodemográficos foram selecionados para o presente estudo e detalhamento sobre as análises espaciais. Adicionalmente, os revisores destacam a interpretação mais adequada do Mapa de Evidências das práticas integrativas
- Resultados - ajustes foram sugeridos nos títulos das tabelas e na configuração dos gráficos. Adicionalmente, na tabela 2 é necessário substituir o termo “associação” por “correlação”, pois foi a análise realizada pelos autores. Especificamente na Tabela 2, as colunas referentes à “Tendência” e “significância” poderiam ser suprimidas. Citar o nível de significância em métodos.
- Discussão - revisão das interpretações dos resultados, de forma a deixá-las mais contextualizadas aos resultados apresentados. Discussão mais direta a respeito da subnotificação no SISAB, quais os vieses que podem estar presentes na análise e as possíveis decisões dos autores para minimizá-los. Revisão da interpretação da correlação encontrada entre o uso das PICS e a faixa etária. A partir do p-valor, não há correlação. Adicionalmente, o termo “incidência” empregado na discussão talvez não seja o mais adequado, pois os resultados são provenientes somente de um ano e refletem o registro (ocorrência) do uso das PICS e da HAS.

Em todo o corpo do manuscrito, é necessário revisar a formatação de acordo com as normas da RESS (disponível em: https://ress.iec.gov.br/p/page/2/instrucoes). Destaco a necessidade de adicionar como última palavra-chave o desenho do estudo.

Todos os itens presentes nos pareceres devem ser respondidos ponto a ponto em uma carta resposta e todas as alterações no texto devem ser destacadas em amarelo.

Recommendation: Major revision 

## Second round comments

Recebemos a versão revisada do artigo intitulado “Práticas Integrativas e Complementares, hipertensão arterial e indicadores sociais no Brasil no ano de 2023: estudo ecológico com análise espacial”. A grande maioria das modificações solicitadas pelos revisores e pelo editor associado foi acatada. Entretanto, algumas correções ainda são necessárias, as quais estão destacadas por seção do artigo. As recomendações de correções foram organizadas em minor e major revisions.

Major revisions

Resumo

Métodos: Citar análises estatísticas realizadas

Resultados: 1) especificar o que foi maior entre as mulheres pela análise de variância (PICS? HAS?) (A Análise de Variância apresentou valores da estatística F com maior utilização entre mulheres...); 2) pelo valor de p não houve diferença segundo a faixa etária. Retirar a interpretação dos resultados terem apresentado “apenas tendência estatística”. Destacar no resumo que não houve diferença segundo a idade.

Métodos

Página 3, primeiro parágrafo - descrição dos dados de HAS do SISAB - foi selecionado “diagnóstico de hipertensão arterial” do SISAB? Descrever exatamente qual o indicador selecionado para a análise

Página 3, terceiro parágrafo (análise de dados) - teste de correlação de Pearson - este teste visa analisar “correlação” e não “associação”, conforme descrito no texto. Ajustar

Página 3, terceiro parágrafo (análise de dados) - detalhar a razão do uso da Análise de Variância - comparação de médias (médias de quais indicadores?)

Discussão

As considerações sobre a revisão de alguns pontos na discussão com relação à interpretação dos resultados não foram acatadas pelos/as autores/as. Tais solicitações são relevantes na discussão, pois refletem a interpretação dos resultados. Os/as autores/as podem fazer uma breve descrição sobre isso na discussão, sem ter necessidade de explicitar com muitos detalhes. Recomendo que tais interpretações sejam realizadas na nova versão a ser apresentada. Destaco abaixo as reflexões realizadas pelo Revisor 3, que não foram acatadas.

“como os autores interpretam tais diferenças? Por exemplo, os estados com maiores taxas de PICS/hab são aqueles com maiores PIB e IDH e vice-versa, ainda que haja exceções, mas o que isto poderia significar? Poderia refletir maior/menor capacidade de contratação de profissionais de saúde habilitados em tais terapias e de compra de insumos para as mesmas? Seria maior/menor conhecimento e formação em tais práticas nesses estados? Ou parte dessas diferenças regionais poderiam ser explicadas por problemas no registro de tais ações ou do atendimento para HA no Sistema segundo o estado federativo? Ademais, as diferenças entre o tipo de PICS segundo estado pode refletir diferenças na oferta devido à disponibilidade de profissionais/insumos, formação e aspectos culturais a depender da PICS.”

Colocar as limitações no segundo parágrafo da discussão, conforme normas da RESS.

Sugiro retirar a limitação “A análise transversal impossibilita a identificação de tendências ao longo do tempo e a determinação de relações causais entre o uso das PICS e o controle da hipertensão arterial.”, uma vez que o objetivo do estudo foi fazer uma análise em um único período.

Citar o mapa de evidências ao relacionar a aromaterapia à hipertensão arterial, conforme sugestão do Revisor 3. (“A aromaterapia foi descrita como capaz de reduzir significativamente a pressão arterial, promovendo relaxamento e vasodilatação, fornecendo assim uma alternativa não farmacológica para o controle da hipertensão (26)”. Importante fazer referência aqui ao mapa de evidências ou diretamente os estudos de revisão sistemática ao invés da referência à apenas um estudo clínico.

Página 6, último parágrafo: pelas análises apresentadas na Tabela 2 não há diferença do uso de PICS segundo a idade das pessoas. Desta forma, rever a interpretação apresentada (“Outro achado importante foi a ocorrência do uso de PICS relacionadas à idade....”)

Ao longo de métodos, resultados e discussão: substituir o termo “associação” por “correlação” quando se referir aos dados que foram analisados pela correlação de Pearson.

Ao longo do manuscrito: remover siglas não consagradas, como as PICS (conforme normas da RESS)

Minor revisions

Resumo

Objetivo: Substituir “avaliar” por “analisar” para se alinhar ao objetivo descrito na introdução do manuscrito Manter somente “estudo ecológico”, retirar o termo “transversal”

Resultados

Citar “SISAB, 2023” nos títulos das tabelas 1 e 2.

Possivelmente, a solicitação do envio das figuras editáveis ocorrerá no processamento do manuscrito, caso ele seja aceito. Desta forma, sugiro aos/as autores/as acatarem essa solicitação.

Recommendation: Major revision 

## Third round comments

Recebemos a versão revisada do artigo intitulado “Práticas Integrativas e Complementares, hipertensão arterial e indicadores sociais no Brasil no ano de 2023: estudo ecológico com análise espacial”. As modificações solicitadas pelo editor associado foram acatadas. Três aspectos importantes necessitam de ajustes, os quais estão listados abaixo. Por gentileza, destacar em amarelo as correções realizadas na próxima versão do manuscrito.

Major revisions

1) Resumo

Objetivo: pelos objetivos apresentados no final da introdução e no primeiro parágrafo de métodos, há o foco principal em correlacionar o uso de algumas práticas integrativas com os atendimentos de hipertensão arterial. Adicionalmente, o uso de práticas integrativas e atendimentos de hipertensão arterial com indicadores socioeconômicos. Desta forma, esse objetivo central deve ser destacado no objetivo do resumo. Sugestão de redação: “Correlacionar o uso de práticas integrativas e os atendimentos de hipertensão arterial, bem como sua correlação com indicadores socioeconômicos”

Métodos: citar os indicadores utilizados antes dos testes estatísticos e descrever o uso dos testes (ex: a correlação de Pearson para correlação entre HAS e práticas integrativas...)

Conclusão: responder aos objetivos - há correlação entre práticas integrativas e atendimento de hipertensão e com indicadores sociodemográficos?”

2) Métodos e Resultados

Rever a descrição em resultados “...teste ANOVA demonstrou diferenças significativas no uso de práticas integrativas e complementares por sexo (estatística F=15,87; p-valor <0,001), com taxas médias substancialmente maiores entre mulheres (60,69/100 mil) versus homens (16,92/100 mil), conforme confirmado pelo post-hoc de Tukey.”

Como o teste estatístico já foi citado em métodos, talvez não seja necessário repeti-lo nos resultados. Um ponto de dúvida: o Teste de Tukey é utilizado para identificar diferenças entre grupos, quando há mais de duas categorias. No caso de sexo, só tem duas categorias. Desta forma, pelo resultado da ANOVA, já é possível verificar se as médias são diferentes entre homens e mulheres. Ajustar o texto.

Pela descrição dos objetivos, há a compreensão de que a correlação entre atendimento de hipertensão arterial e os indicadores socioeconômicos também seria realizada, mas não há essa análise. Talvez fosse interessante realizá-la. Acredito que esse resultado apoia a interpretação das desigualdades regionais.

3) Discussão

No terceiro parágrafo, na sentença “Todavia, mesmo com as limitações apontadas anteriormente, a análise revelou importantes disparidades na adoção dessas práticas, com maior prevalência em estados com melhores condições de IDHM e PIB per capita, além de uma maior adesão entre idosos, grupo frequentemente afetado por condições crônicas.”, sugiro retirar esse resultado que foi superior nos idosos, pois pela análise estatística, não teve diferença.

Limitações: “Além disso, o estudo foca apenas na atenção primária, desconsiderando práticas realizadas em outros níveis de atenção do SUS e no setor conveniado ao SUS.”. Se os autores tiverem algum resultado das estimativas do uso das práticas integrativas em locais diferentes da atenção primária seria interessante apresentar, para verificarmos se a concentração do uso é maior ou menor do que na atenção básica.

Recommendation: Major revision 

## Fourth round comments

Recebemos a versão revisada do artigo intitulado “Práticas Integrativas e Complementares, hipertensão arterial e indicadores sociais no Brasil no ano de 2023: estudo ecológico com análise espacial”. Agradecemos pelas correções realizadas. As modificações solicitadas pelo editor associado foram acatadas e considero o manuscrito aceito. Abaixo destaco duas pequenas alterações no resumo e no método. Parabenizamos pelo manuscrito.

1) Resumo

Objetivo: Substituir o termo “associação” por “correlação”, para corresponder exatamente ao que foi realizado. Sugestão de redação: “Correlacionar o uso de práticas integrativas e os atendimentos de hipertensão arterial, bem como sua correlação com indicadores socioeconômicos”

Métodos (análises): Sugestão de redação “Foram descritas as taxas de PICs e hipertensão, utilizado o coeficiente de correlação de Pearson.....”

2) Métodos:

Substituir “teste de correlação de Pearson” por “Coeficiente de Correlação de Pearson” .

Atenciosamente,

Recommendation: Accept 

